# The Stability Analysis Method of the Cohesive Granular Slope on the Basis of Graph Theory

**DOI:** 10.3390/ma10030240

**Published:** 2017-02-27

**Authors:** Yanpeng Guan, Xiaoli Liu, Enzhi Wang, Sijing Wang

**Affiliations:** 1 State Key Laboratory of Hydro Science and Engineering, Tsinghua University, Beijing 100084, China; hbgyp2008@163.com (Y.G.); nzwang@tsinghua.edu.cn (E.W.); wangsijing@tsinghua.edu.cn (S.W.); 2 Institute of Geology and Geophysics of the Chinese Academy of Sciences, Beijing 100029, China

**Keywords:** stability analysis, progressive failure, slope stability, DEM (discrete element method), particle, macro-contact

## Abstract

This paper attempted to provide a method to calculate progressive failure of the cohesive-frictional granular geomaterial and the spatial distribution of the stability of the cohesive granular slope. The methodology can be divided into two parts: the characterization method of macro-contact and the analysis of the slope stability. Based on the graph theory, the vertexes, the edges and the edge sequences are abstracted out to characterize the voids, the particle contact and the macro-contact, respectively, bridging the gap between the mesoscopic and macro scales of granular materials. This paper adopts this characterization method to extract a graph from a granular slope and characterize the macro sliding surface, then the weighted graph is analyzed to calculate the slope safety factor. Each edge has three weights representing the sliding moment, the anti-sliding moment and the braking index of contact-bond, respectively, $E_{1}E_{2}E_{3}E_{1}E_{2}E_{3}$. The safety factor of the slope is calculated by presupposing a certain number of sliding routes and reducing Weight $E_{3}$ repeatedly and counting the mesoscopic failure of the edge. It is a kind of slope analysis method from mesoscopic perspective so it can present more detail of the mesoscopic property of the granular slope. In the respect of macro scale, the spatial distribution of the stability of the granular slope is in agreement with the theoretical solution.

## 1. Introduction

Slope engineering is common in geotechnical engineering and most of the slopes are formed by rock blocks or soil clusters. Many researchers have attempted to develop the slope stability analysis method. Among these methods, the limit equilibrium methods (LEM) are widely used. The safety factor in this method is defined as the quotient of the anti-sliding force divided by the sliding force on the critical sliding surface [1].

Some numerical methods such as finite element method (FEM) and numerical manifold method (NMM) can obtain more accurate stress–strain state of the model. They get the safety factor by increasing the acceleration of gravity or reducing the macro strength parameters (such as the cohesion and internal friction angle) [2]. The increase/reduction coefficient is defined as the safety factor but its correlation with the safety factor in limit equilibrium method is still in debate [3].

Some researchers adopted graph theory to analyze the numerical results of the slope engineering to get the safety factor [4,5]. Li et al. combined graph theory with FEM to analyze the slope stability and obtained good results that are comparable to limit equilibrium method [4]. Zheng et al. combined graph theory with NMM to calculate the safety factor of the slope with discontinuous fractures by clever designs of the graph [5]. Most numerical methods (such as FEM, NMM and Meshless Method) have common properties that they adopt macro strength parameters such as the internal angle and cohesion to define the strength of the materials.

Another numerical method is Discrete Element Method (DEM), which has been applied to slope engineering as a method of discontinuous mechanics [6,7,8,9]. Its main characteristic is that it adopts mesoscopic parameters of the particles instead of the macro parameters of the materials to construct its constitutive model. The relation between the mesoscopic parameters and the macro parameters can be calibrated by virtual biaxis test in DEM. The advantage of DEM is that it simulates the heterogeneity of granular materials by the random configuration of the particles, which is closer to the property of the granular material. Therefore, the slope analysis in DEM is necessary for further research on the property of the granular slope. However, its entirely different constitutive model makes some previous analysis methods unfit for DEM.

Wang et al. used PFC2D to analyze the stability of heavily jointed rock slopes and found that the failure mode of the slope in heavily jointed rock depends on the joint quantity [6]. He et al. studied the seismic response of colluvium accumulation slope by particle flow code and revealed that the presence of surficial accumulation is the major controlling factor of ground-motions around the bed rock [7]. Camones et al. studied the step-route failure mechanism in rock slope and calculated the safety factor by increasing acceleration of gravity [8]. Huang et al. studied the step-route failure of rock slopes with intermittent joints and deduced the computational method of the safety factor of the rock slope [9]. Most slope analysis methods except DEM adopt macro strength parameters to define their safety factor so the safety factor of the slope in DEM should be further discussed. Moreover, most researches using DEM adopt reducing the strength parameter or increasing the acceleration of gravity as the slope analysis method, which can only get the critical slip surface. The spatial distribution of the stability need further research.

The aim of this paper is to get the safety factor of granular slope and present the spatial distribution of the stability. The methodology can be divided into two parts. Section 2 states Part 1, which is the characterization method of macro-contact on the basis of graph theory. Section 3 elaborates Part 2, which is the detailed method of calculating the contact force on the macro contact surface and analyzing the safety factor of the granular slope on the basis of Part 1. Section 4 is a case study and the corresponding result analysis. Section 5 reports the comparison of the results obtained in this study with those from the literature or obtained based on different methods. The Conclusion Section is provided at the end of the paper.

## 2. The Characterization Method of Macro Contact in Two Dimensional DEM

### 2.1. The Characterization Method of the Particle Contact

**Figure 1:**
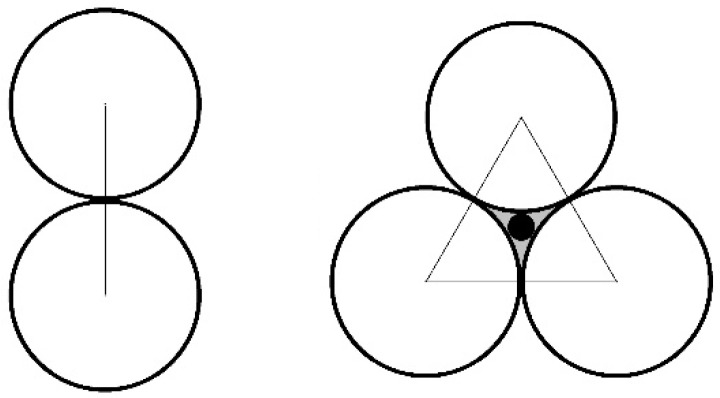
The particle-connection and the void cell.

In this paper, substances of the granular material are not infinitely divisible. One particle is the minimum element of the granular material. Particle contact is the particle–particle contact. As shown in Figure 1, Particle-connection is defined as the connecting line of two adjacent particle centers. Specific particle-connections from end to end can form a closed structure in which there is an intergranular void. Void cell is defined as the internal space of this closed particle-connection chain. There is a black dot in the center of the void cell that can characterize it [10,11,12].

**Figure 2:**
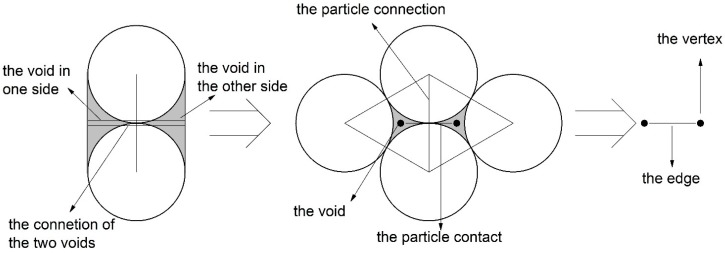
The characterization method of the particle contact and the abstracted graph.

Since a certain number of particle-contacts form the macro-contact surface, the particle-contact is a contact surface with a certain length rather than contact point. As shown in Figure 2, the particle contact can be treated as the coalescence of the two voids located on the two sides of particle-connection. Since the void cell mentioned above can characterize the void, the connecting line of centers of the adjacent void cells can characterize the particle contact. 

As shown in Figure 2, based on graph theory, a graph G=(V,E) is created to characterize the particle contact between the particles. The void cells are characterized by the vertex set V of the graph, which is shown as black dots. The particle contacts shared by adjacent void cells are characterized by the edge set, which is shown as black straight lines. The edge e can be assigned to a certain number of weights which is recorded as w(e). Examples include the contact strength, contact force, etc.

### 2.2. The Characterization Method of the Macro-Contact

**Figure 3:**
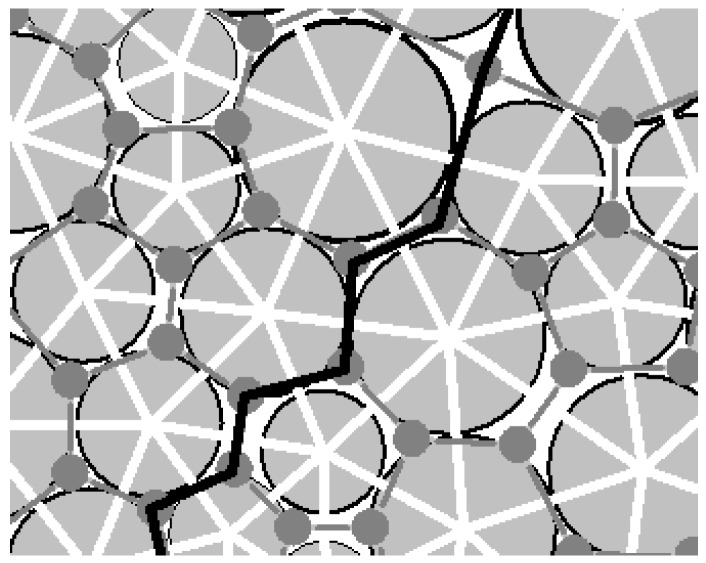
The particle-connection, the void cell and the particle contact in the aggregation of particles with different radius.

The aggregation of particles with different radiuses is shown in Figure 3. Gray circles characterize the particles and white lines indicate the particle-connections of the adjacent particles. This closed white line and its internal space are just the void cell mentioned above. The gray dot is the center of the void cell and the gray lines are the connecting lines between the adjacent gray dots.

In the two-dimensional case, the definition of the macro-contact can be stated in two aspects: physics and topology. In physics, it is composed of a certain number of particle contacts. In topology, it is a 1-manifold (short for one-dimensional manifold) with boundary. (A 1-manifold can be interpreted intuitively as a curve that does not intersect itself.) Therefore, the macro-contact is a particle-contact sequence which satisfies the constraint condition of 1-manifold. As the black lines in Figure 3 show, the particle-contact sequence in which the particle contacts connect each other from end to end by pointing to the same voids can satisfies the constraint condition of 1-manifold. Therefore, the macro-contact can be characterized by the edge sequence in which the edges connect each other in form of end-to-end.

## 3. The Computing Method of the Slope Stability

### 3.1. A Simple Introduction to DEM

The principle of the DEM has been extensively described in other papers [13,14], so this paper will briefly describe the key features of DEM used in this study [15].

A contact-stiffness model can provide a relation between the normal ($F_{n}$) and shear ($F_{s}$) components of contact forces and the relative displacements ($U_{n}$, $U_{s}$). The simplest model is the linear model. Assuming two particles A and B are in contact, their normal stiffness values are $K_{n}^{A}$ and $K_{n}^{B}$ and shear stiffness are $K_{s}^{A}$ and $K_{s}^{B}$, respectively. The normal and shear stiffness of the contact can be computed by the following equations:

(1) $$K_{n}=\frac{K_{n}^{A}\cdot K_{n}^{B}}{K_{n}^{A}+K_{n}^{B}}$$

(2) $$K_{s}=\frac{K_{s}^{A}\cdot K_{s}^{B}}{K_{s}^{A}+K_{s}^{B}}$$

The force–displacement law of two particles in contact is as follows. $F_{n}$ is the normal contact force. $F_{s}$ is the shear contact force. $U_{n}$ is the normal overlap between the two particles in contact. $U_{s}$ is the tangential overlap.

(3) $$F_{n}=K_{n}\cdot U_{n}$$

(4) $$\Delta F_{s}=K_{s}\cdot \Delta U_{s}$$

**Figure 4:**
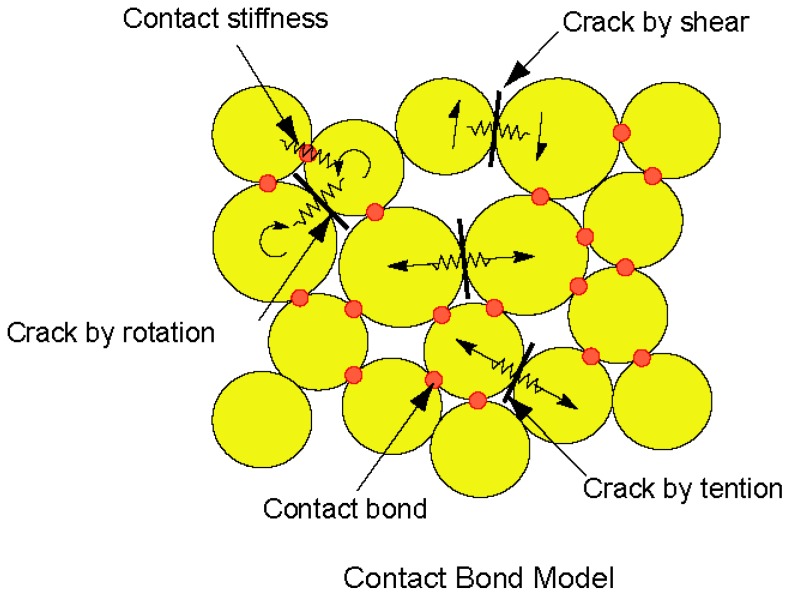
Illustration of bond models provided in PFC (Particle Flow Code) [16].

As shown in Figure 4, the bond-contact model is adopted in this study. If the tensile stress (shear stress) at a contact exceeds the normal bond strength NBS (shear bond strength SBS), the bond will break and separation or frictional sliding can occur.

DEM has a significant property that its parameters are mesoscopic, which are different from the macro parameters in other numerical methods. For example, the friction in FEM is a macro friction and it can be regarded as the macro effect of the mesoscopic friction on the particle surface and the engagement force between the particles in contact. Therefore, the slope analysis method in DEM should be further discussed. The following is the detailed analysis method.

### 3.2. The Detailed Method to Characterize the Sliding Surface

**Figure 5:**
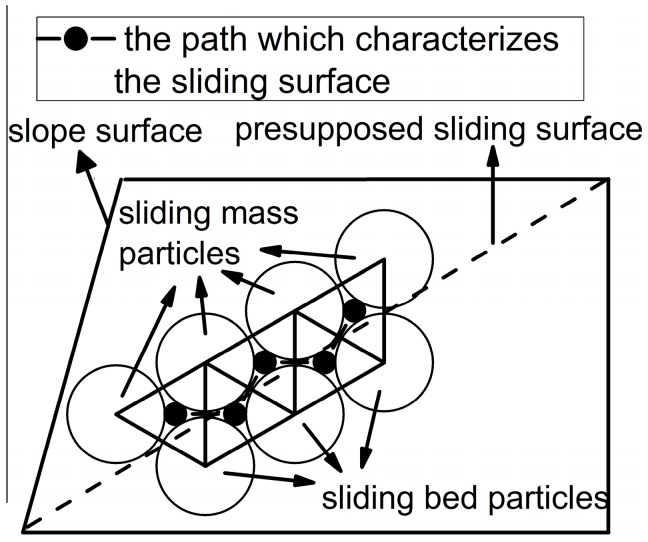
The particle contacts which characterize the sliding surface.

In the slope project, sliding surface is a kind of the macro-contact mentioned above. Therefore, the characterization method of macro-contacts can characterize the sliding surface of the granular slope. As shown in Figure 5, the edge sequence in the form of end-to-end which is similar to the corresponding sliding surface in terms of the route can be found to characterize the sliding surface. Due to the randomness of granular materials, the edge sequence is not just right on the presupposed sliding surface but basically near the sliding surface. The edge sequence can approximately characterize the presupposed sliding surface and it will separate all particles into sliding mass particles and sliding bed particles.

### 3.3. The Computing Method of the Cohesive Soil Slope Stability

When the soil slope with the characteristic of strain softening slides, the stress of the soil near the sliding surface does not reach the shear strength at the same time. When the stress of the soil located at a certain place reaches the shear strength, the shear failure occurs at this place and corresponding peak strength will become residual strength. At the same time, failure will extend to other places. If the shear failure occurs through a route from slope toe to slope crest, the slope will slide integrally. That is the whole progress of progressive landslide. The aim of this article is to simulate this progress in graph theory.

In the characterization method of the contact surface, the vertexes of a certain graph characterize the void cell and the edges characterize the particle contacts of the granular material. The vertexes in the slope toe are defined as vertex group $V_{a}$ and the vertexes in the slope crest are defined as vertex group *V*_b_. There are many routes from vertex group $V_{a}$ to vertex group $V_{b}$ and each route has a corresponding safety factor. Among all the whole safety factors, the minimum safety factor is the global safety factor of the slope and its corresponding route is the most dangerous sliding surface of the slope. This method can be described as follows: given a weighted directed graph *G* = (*V*,*E*), the vertexes in the slope toe are defined as vertex group $V_{a}$ and the vertexes in the slope crest are defined as vertex group $V_{b}$. The sliding surface is the route which corresponds to the minimum safety factor among all the possible routes from vertexes $V_{a}$ to vertexes $V_{b}$.

There are many routes that need to be analyzed. In order to simplify the problem and improve calculation speed, a certain number of sliding surfaces are supposed. Referring to the slip circle method, a certain number of circle centers are supposed and every circle center responds to multiple sliding surfaces with different radiuses. Referring to the characterization method above, the edge sequence, which is similar to the route of the sliding face, can be calculated. Each edge of the edge sequence has three weights. Weight $E_{1}$ is the sliding moment of the contact and weight $E_{2}$ is the anti-sliding moment of the contact. The safety factor of a certain edge sequence is calculated by reducing weight $E_{2}$ repeatedly and this safety factor is stored in the vertexes forming this edge sequence. One vertex can be the component of a certain number of edge sequences, so one vertex can have many safety factors and the entire safety factor of the vertex are the minimum of these safety factors. Weight $E_{3}$ is contact-bond broken flag and value 1 means that a contact bond was present at the corresponding contact in the past but has been judged to be broken.

**Figure 6:**
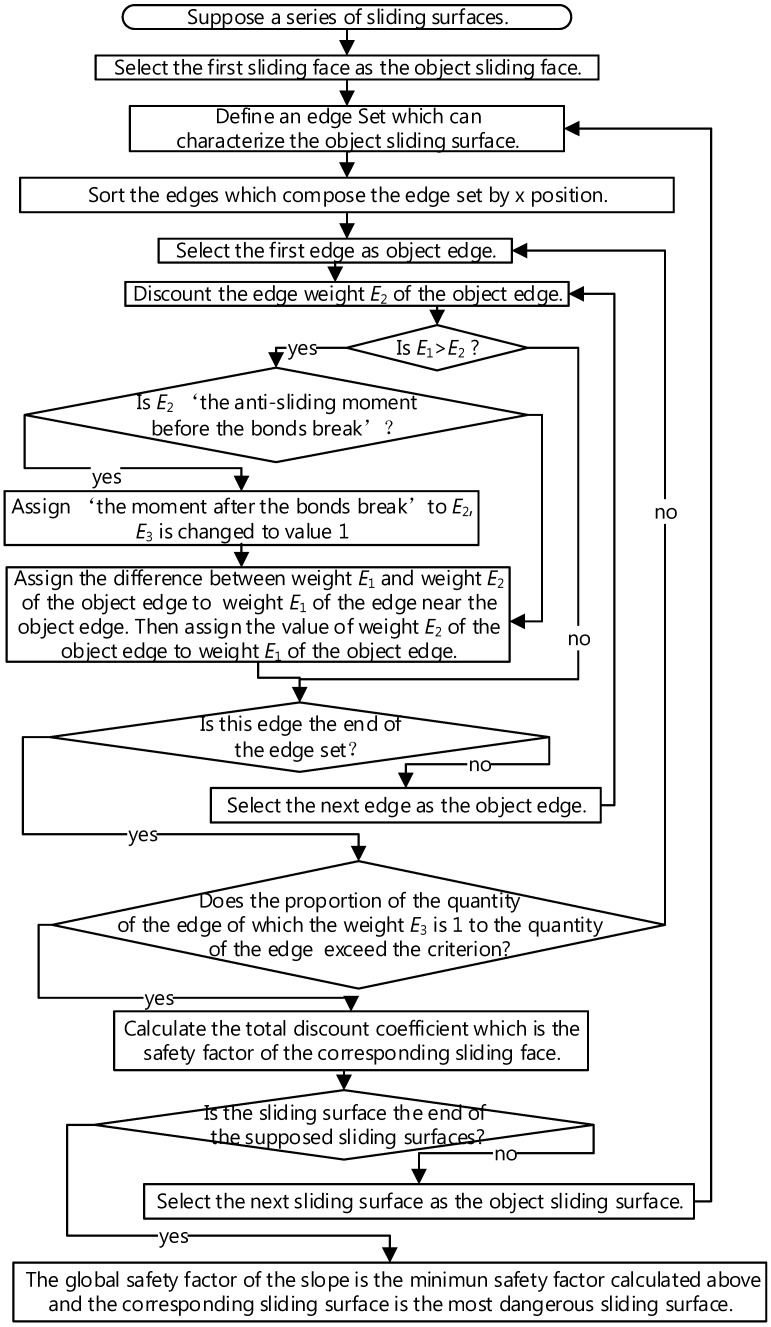
The progress of computing the cohesive soil slope stability.

The detailed calculation method is shown in Figure 6. In the graph *G*, weight $E_{2}$ should be classified into two types: the anti-sliding moment before the corresponding contact bond break (before softening) and the anti-sliding moment after the corresponding contact bond break (after softening). Most weights of edge $E_{2}$ are the anti-sliding moment before the corresponding contact bond breaks. If weight $E_{2}$ is reduced to a certain extent, weights $E_{1}$ of the corresponding contact may exceed the weight $E_{2}$, then the bond of the corresponding contact is judged to break and weight E_3_ is changed to value 1 from value 0. The corresponding weight $E_{2}$ is changed to the anti-sliding moment after the corresponding contact bond breaks. At this moment, weight $E_{1}$ and weight $E_{2}$ cannot maintain the balance but the slope may not slide integrally. The part of weights $E_{1}$ which cannot be balanced by the corresponding weights $E_{2}$ will be balanced by weights $E_{2}$ of other adjacent edges. If the ratio of the edges of which weight $E_{3}$ is value 1 to the number of the whole edges forming the edge sequence exceeds a certain extent, the slope is determined to slide and the corresponding reduction factor is the safety factor of the corresponding sliding surface. Among the safety factors of all the supposed sliding surfaces, the minimum safety factor is the entire safety factor of the slope and the corresponding surface is the most dangerous sliding surface. The safety factors of all the location can be shown by color mapped figure.

### 3.4. The Computing Method of the Edge Weight

The parameters adopted to calculate the safety factor of the granular slope are given in Table 1.

**Figure 7:**
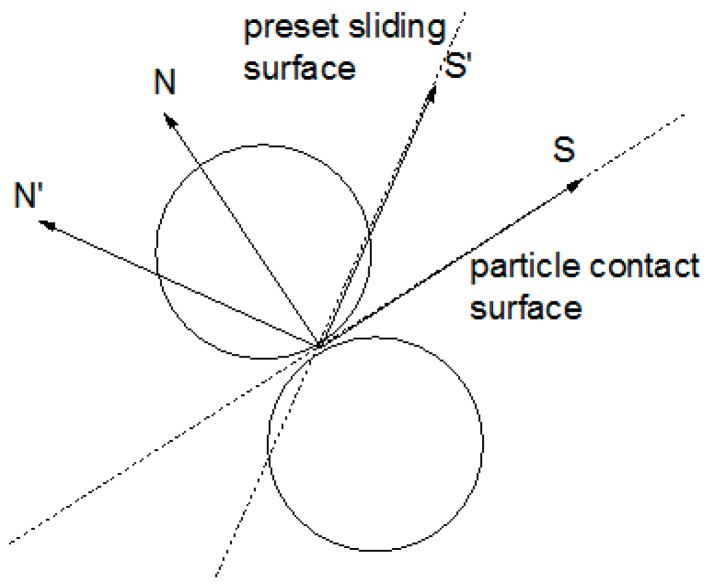
The direction of preset sliding surface and particle contact surface.

As shown in Figure 7, the particle contact surface may not overlap the preset sliding surface. The direction $S^{\prime }$ and $N^{\prime }$ are the tangential direction and the normal direction of preset sliding surface, respectively. The direction S and N are the tangential direction and the normal direction of particle contact surface, respectively.

$F_{s^{\prime }}$ is the projection of the contact force in the tangential direction of preset sliding surface. $F_{n^{\prime }}$ is the projection of the contact force in the normal direction of preset sliding surface.

(5) $$F_{s^{\prime }}=F_{c}\cdot S^{\prime }$$

(6) $$F_{n^{\prime }}=F_{c}\cdot N^{\prime }$$

$F_{s}$ is the projection of the contact force in the tangential direction of particle contact surface. $F_{n}$ is the projection of the contact force in the normal direction of particle contact surface.

(7) $$F_{s}=F_{c}\cdot S$$

(8) $$F_{n}=F_{c}\cdot N$$

In the characterization of the contact surface of the granular material, according to the constitutive model of the granular material, the weight $E_{1}$ (sliding moment) of the edge is the moment of the contact force located at the contact characterized by the corresponding edge about the supposed slip circle center.

(9) $$E_{1}=(F_{c}\times N^{\prime })DIS$$

As mentioned above, the calculation method of the weight *E*_2_ should be classified into the anti-sliding moment before the corresponding contact bond break and the anti-sliding moment after the corresponding contact bond break. There are two types of bond strength, contact bond normal strength and contact bond shear strength.

The calculation method of the anti-sliding moment before the contact bond break is relative to its stress state, which can be expressed as follows: for particles of which the contact force type is compression, the failure type is likely to be shear dislocation when the shear force reaches the contact bond shear strength. This is because particles are inseparable and cannot be crushed. $Q_{s}$ is defined as the quotient of the contact bond shear strength divided by the corresponding shear force and the anti-sliding moment is the product of the sliding moment multiplied by $Q_{s}$.

(10) $$Q_{s}=\frac{SBS}{F_{s}}$$

(11) $$E_{2}=E_{1}Q_{s}$$

For particles of which the contact force type is tension, there may be two kinds of failure types, shear dislocation and tension fracture. If the shear force reaches the contact bond shear strength or the normal force reaches the contact bond normal strength, the bond will break. Thus, $Q_{s}$ is defined as the quotient of the contact bond shear strength divided by the corresponding shear force and $Q_{n}$ is defined as the quotient of the contact bond normal strength divided by the corresponding normal force. The $Q_{\min}$ is defined as the smaller one between $Q_{s}$ and $Q_{n}$. The corresponding anti-sliding moment is the product of the sliding moment multiplied by $Q_{\min}$.

(12) $$Q_{n}=\frac{NBS}{F_{n}}$$

(13) $$Q_{min}=\min\left(Q_{t},Q_{n}\right)$$

(14) $$E_{2}=E_{1}Q_{min}$$

For particles of which the contact force type is compression, the calculation method of the anti-sliding moment after the contact bond break is the moment of the macro friction about the sliding circle center, which can be calculated by the following equation.

(15) $$E_{2}=\left(f\times N^{\prime }\right)DIS$$

For particles of which the contact force type is tension, the adjacent particles are likely to separate from each other and there may be no friction between the adjacent particles after the corresponding bond breaks, so the anti-sliding moment is zero.

(16) $$E_{2}=0$$

Therefore, the computational formula of $E_{2}$ can be expressed as follows:

(17) $$E_{2}=\{\begin{array}{c} E_{1}Q_{t}\;\mathrm{before}\;\mathrm{the}\;\mathrm{bond}\;\mathrm{breaks},\;F_{n}>0\\ E_{1}Q_{min}\;\mathrm{before}\;\mathrm{the}\;\mathrm{bond}\;\mathrm{breaks},\;F_{n}<0\\ \begin{array}{c} \left(f\times N^{\prime }\right)DIS\;\mathrm{after}\;\mathrm{the}\;\mathrm{bond}\;\mathrm{breaks},\;F_{n}>0\\ 0\;\mathrm{after}\;\mathrm{the}\;\mathrm{bond}\;\mathrm{breaks},\;F_{n}<0 \end{array} \end{array}$$

## 4. Case Study and the Corresponding Result

### 4.1. Case Study

**Figure 8:**
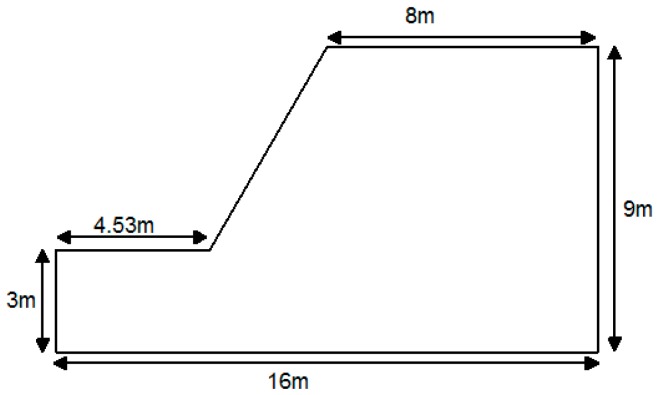
The geometry profile of the slope example.

**Figure 9:**
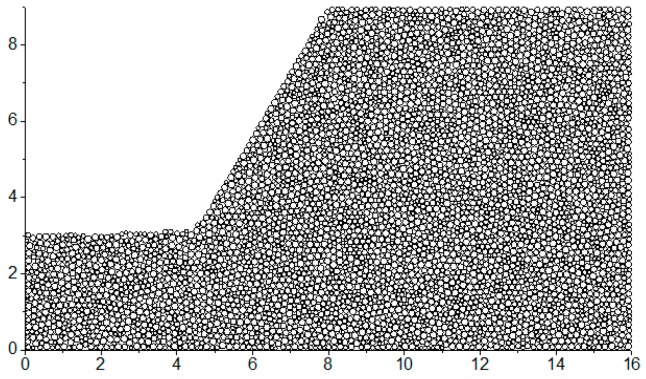
The granular slope example.

A numerical model is built to help state the computing method of slope stability on the basis of characterization method of macro-contact. The slope height of the example is 6 m and the corresponding slope angle is 60°. The detailed geometry profile of the example and the corresponding model composed of granular material are shown in Figure 8 and Figure 9, respectively. The model is constructed by the expansion of the random particles inside the slope boundaries. The particles in the slope top do not touch the boundary in the top in case there is additional loads on the slope top. The macro parameters and the corresponding mesoscopic parameters of the example are shown in Table 2 and Table 3, respectively. The mesoscopic parameters are determined by the calibration in the virtual biaxial compression test in DEM [17]. Firstly, the virtual biaxial compression tests with different mesoscopic parameters should be done. Then, the macro mechanical responses of the simulated results will be compared with the macro parameters of the material. Finally, the mesoscopic parameter that can present the macro mechanical responses consistent with the material property could be selected to simulate the material.

**Figure 10:**
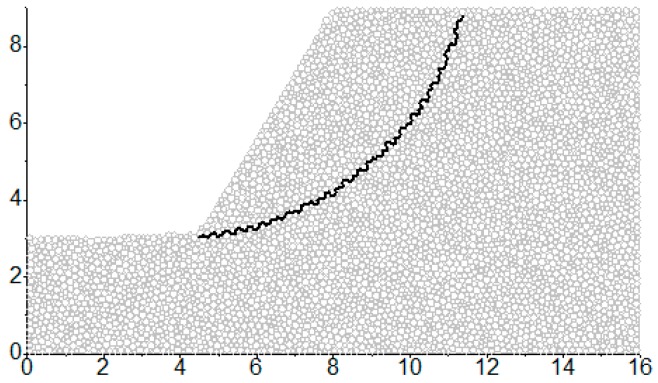
The edge sequence which characterizes the sliding surface.

**Figure 11:**
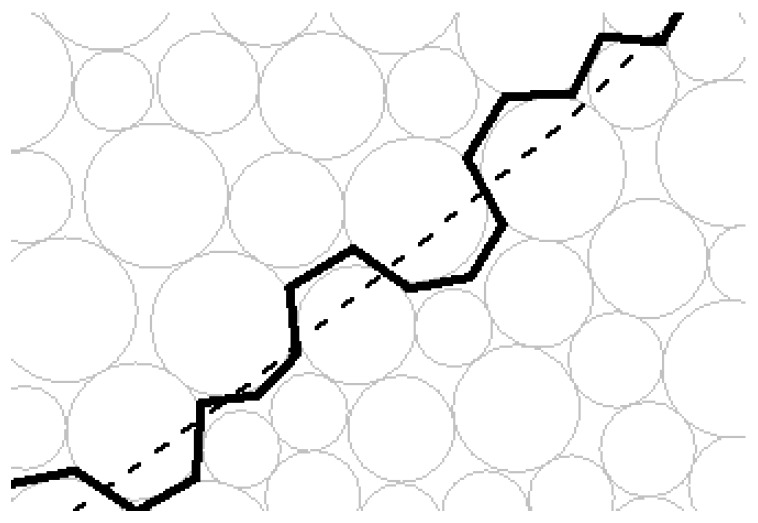
The enlarged drawing of the sliding surface and the corresponding edges characterizing it.

At first, a series of sliding surfaces can be preset. For the simplification of the calculation, the circular sliding surface is adopted. The sketch map is shown in Figure 10 in which the coordinate of circle center in Figure 10 is (3.8, 10.8) and the corresponding radius of sliding surface is 7.8 m. As shown in Figure 11, due to the randomness of granular materials, the particle contact is not necessarily on the sliding surface, but basically near the sliding surface.

### 4.2. The Detailed Parameters

There are two adjustable parameters in the computing method of the slope stability mentioned above: the impact load caused by the bond break and the computing method of macro friction. The following is the discussion of the value of these adjustable parameters. 

#### 4.2.1. The Impact Load Caused by the Bond Break

**Figure 12:**
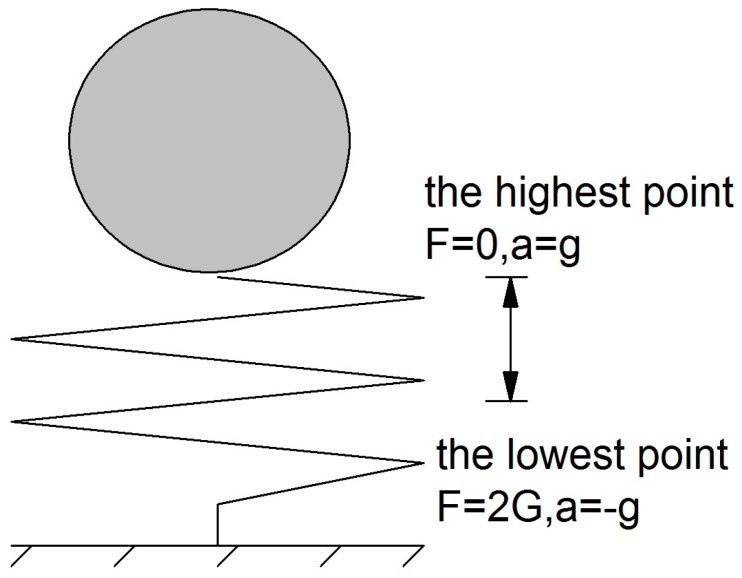
A weight with no velocity is placed above a spring suddenly.

When the contact bond breaks, the sliding moment that is originally undertaken by the anti-sliding moment at this contact will be undertaken by the anti-sliding moment of its adjacent contacts suddenly. Therefore, the load undertaken by its adjacent contact is an impact load. The slope studied in this article is cohesive soil slope. Its failure mode is mostly brittle failure and the particle is arranged closely, so the velocity of the particle is comparatively low before the sliding mass start to slide. As shown in Figure 12, if a weight with no velocity is placed above a spring suddenly, the weight will be in situation of simple harmonic vibration and the contact force will reach maximum when the particle reaches the lowest point. In Figure 12, F is the contact force between the weight and the spring, a is the accelerated speed and g is the acceleration of gravity. The max contact force is the double of the particle weight. Therefore, when weight $E_{2}$ is reduced to the extent that the bond of the corresponding contact is judged to break, the part of weights $E_{1}$ which cannot be balanced by the corresponding weights $E_{2}$ will be doubled and then transferred to be undertaken by weight $E_{2}$ of its adjacent edges. No matter the bond of these adjacent edges is judged to break under the impact load or not, the doubled load will convert back to the original load after this impact. 

#### 4.2.2. The Friction

The calculation method of the anti-sliding moment after the contact bond break is the moment of the macro friction about the sliding circle center. The macro friction has two parts: the mesoscopic friction on the particle surface and the engagement force between the particles in contact. Since the macro friction in this computing method is a kind of macro effect, it is not appropriate to select the mesoscopic friction on the particle surface for calculating the anti-sliding moment. The friction adopted in this article can be get in the following equation:

(18) $$f=\tan\varphi F_{n}^{\prime }S^{\prime }$$

$F_{n}^{\prime }$ is the normal force in the preset sliding circle surface. $S^{\prime }$ is the tangential direction of the preset sliding circle surface. tanφ is obtained from the calibration of the macroscopic parameter from virtual biaxial test in DEM. Therefore, the computing method of macro friction f is consistent with the limit equilibrium method.

### 4.3. The Result

#### 4.3.1. The Failure after Reducing the Strength Parameters

**Figure 13:**
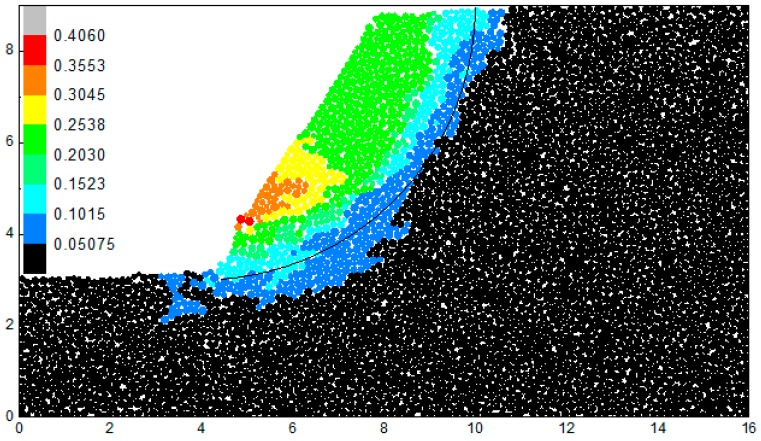
The particle displacement at the 10,000th step after the reduction of mesoscopic parameter.

If the bond strength and friction coefficient is reduced to 72% of supposed value, the slope start to slide. The particle displacement at the 10,000th step after the reduction of the bond strength and the friction coefficient is shown in Figure 13. The bond in the slope toe breaks first, which indicated that it is a retrogressive landslide. The location of the maximal displacement is not precisely the slope toe but about 1 m higher than the slope toe. In order to analyze the slope, the solid line in Figure 13 is marked as the most dangerous slip surface calculated by Janbu method. The circle center of sliding surface A is (4, 9.4) and the corresponding radius is 6.4. It is observed that the displacement of the slope is consistent with the result of Janbu method. The shape of the sliding mass in DEM is consistent of the result of Janbu method. The x coordinate of pull cracks in the slope crest is about 11, which is larger than the x coordinate calculated by Janbu method. The y coordinate of the bottom of the sliding mass is 2.2, which is smaller than the y coordinate calculated by Janbu method.

#### 4.3.2. The Safety Factor

**Figure 14:**
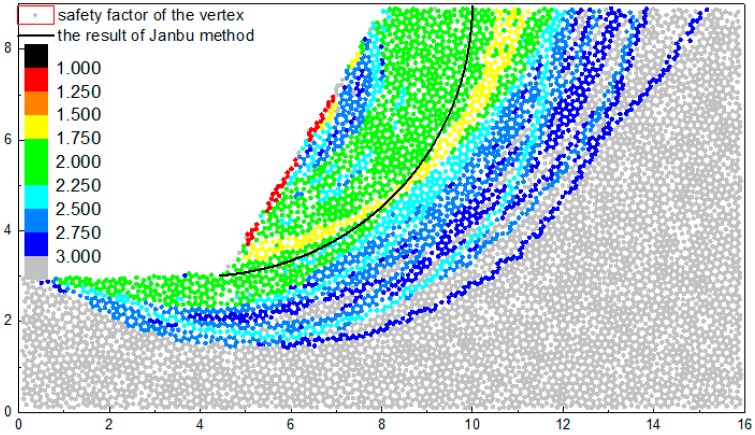
The safety factors of the slope example calculated by the characterization method of the contact surface.

The calculation result of the slope safety factor on the basis of the characterization method of the macro-contact is shown in Figure 14. The result is presented by the color map of the vertex safety factors and the most dangerous sliding surface calculated by Janbu method is also marked in Figure 14. The safety factor of a certain vertex is the minimum of the safety factors of the all the routes formed by this vertex.

In DEM, the configuration of the particles is random, which can present the heterogeneity of granular materials. Therefore, the results will present a degree of heterogeneity and cannot form a continuous contour map like FEM. As is shown in Figure 14, the safety factors of the vertexes are discrete but most of the value of the adjacent vertexes are close in general and can be classified into one band in the color map. For example, the yellow band represents the safety factor value ranging from 1.5 to 1.75. However, due to the heterogeneity of the granular materials, some safety factors of the vertexes may differ from other safety factors signally in the same area and they cannot be classified into one band, which leads to the mixing phenomena in the boundary of two adjacent bands. For example, there are some green vertexes (1.75~2.0) in the yellow band (1.5~1.75). In general, the difference between the bands in mixing does not exceed one level. The result of the method raised in this article can still present the consistency with the theoretical method. The location of the critical sliding surface in this method (yellow band) is adjacent to that of the theoretical method.

The color of some vertexes in the slope surface is red and orange which represents the value between (1.0~1.5), which indicates that some of the particles in the slope surface are unstable and may avalanche. The yellow band (1.5~1.75) represents the critical sliding surface. The minimum safety factor in the yellow band is 1.55, which is a little larger than the safety factor 1.3 calculated by the limit equilibrium method. The location and shape of the yellow band and the result of Janbu method is similar except some small differences. Compared with limit equilibrium method, the location of the slope toe in yellow band is more adjacent to the particles with the maximal displacement and the location of the slope crest in yellow band is more adjacent to the particles with the tension fracture. However, this does not mean that the result calculated by limit equilibrium method is inaccurate. Due to the randomness of particle distribution in DEM, no two DEM runs obtain the same results. The location of the critical sliding surface will change with the configuration of the particles. Therefore, the result presents a certain degree of randomness.

The green band (1.75–2.25) is located in the two sides of yellow band. The scope of the upper one is large and regular, which means that the safety factor in this band increases steadily and the increasing rate is slow. The downside of the green band is narrower than the upside part. Most bands in the downside are cyan (2.25~2.5) and blue (2.5~3.0), which indicate that the safety factor in the downside increases significantly. That is because the particles under the slope toe can resist the sliding.

**Figure 15:**
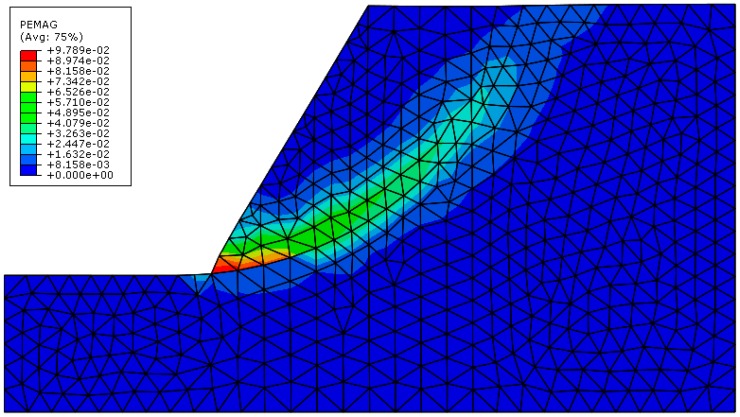
The plastic strain magnitude in the FEM model

The slope is simulated by FEM. According to the result of Li et al. [4], the result calculated by the graph theory in FEM is consistent with the result of strength reduction method. Therefore, the result of this paper is compared with that of strength reduction method in FEM. When the strength factor is reduced to 1/1.47 of the original strength value, the plastic zone crosses the slope section from the slope toe to the slope crest. As is shown in Figure 15, the plastic strain magnitude in the reduced model is shown in figure. The safety factor calculated by FEM is 1.47 which is close to 1.55 calculated by this paper. The location of the critical sliding surface of the two models is also consistent.

#### 4.3.3. The Case Considering Dilatation Softening

**Figure 16:**
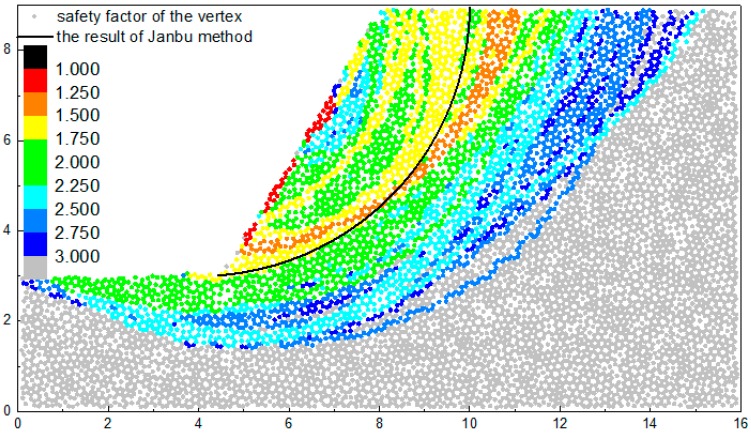
The safety factors on the condition that the internal friction angle is transferred to half of its previous value.

The calculated overall safety factor is a little lager compared with the result of Janbu method. The method in this paper does not consider the impact of dilatation softening. Although the slope is a cohesive slope and its failure mode is mainly brittle failure, the dilatation softening also can have a certain amount impact on its stability. In the later period of the strength reduction, most bonds between the particles in the sliding surface have broken. The property of the particles without bond will be more like the property of coarse-grained soil. The particles without bond can rotate relative to other particles to some extent, which causes the decrease of the internal friction angle. Figure 16 is the safety factors of the slope example on the condition that the internal friction angle is transferred to half of its previous value.

As can be seen in Figure 16, there is an orange band (1.25~1.5) near the result of Janbu method. Its location is very close to the location of the yellow band (1.5~1.75) in Figure 14. That is consistent with the safety factor 1.3 calculated by the limit equilibrium method.

Most yellow band (1.5~1.75) is located on the two sides of the orange band, while a part of the yellow band mixes with the green band (1.75~2.25) due to the heterogeneity of particle accumulation. The region of the green bands on the top of the result of Janbu method shrinks and the region of the green bands under the result of Janbu method is enlarged. The enlarged region is concentrated on the middle slope and slope crest. The cyan band and blue band expand toward the right sides. The change of the cyan band and blue band are small generally, which is relative to its large cardinal number of safety factor.

As a whole, the safety factors of most of the region become smaller compared with the results without considering the dilatation softening. The mixing phenomena still exist in the boundary of two adjacent bands. However, the mixing phenomenon of two bands of which the difference exceeds one level is not common.

## 5. Discussion

### 5.1. The Comparison with Strength Reduction Method of Continuum Method

Traditional limit equilibrium method adopts Mohr–Coulomb yielding criteria. The safety factor is defined as the ratio of shear strength about the shear force in the sliding surface. It can be expressed as the following equation:

(19) $$\omega =\frac{\int_{0}^{l}\left(c+\sigma \tan\varphi \right)dl}{\int_{0}^{l}\tau dl}$$

where ω is the safety factor, σ the normal stress, τ the shear stress, c the cohesion, and φ the internal friction angle. If the two ends of the equation are divided by ω, it is transferred to [3,18]

(20) $$1=\frac{\int_{0}^{l}\left(\frac{c}{\omega }+\sigma \frac{\tan\varphi }{\omega }\right)dl}{\int_{0}^{l}\tau dl}=\frac{\int_{0}^{l}\left(c^{\prime }+\sigma \tan\varphi^{\prime }\right)dl}{\int_{0}^{l}\tau dl}$$

In this equation:

(21) $$c^{\prime }=\frac{c}{\omega }$$

(22) $$\tan\varphi^{\prime }=\frac{\tan\varphi }{\omega }$$

The left side of Equation (19) is 1, which indicates that the slope will reach ultimate limit state when the strength reduces to 1/ω times the previous strength. Therefore, the strength reduction method is consistent with the limit equilibrium method. Since the computing method in this paper adopts the idea of strength reduction method, this conclusion about the reasonability of strength reduction method is appropriate for the computing method in this paper.

### 5.2. The Comparison with Strength Reduction Method of DEM

The strength reduction method of DEM has been discussed and applied to slope engineering. The result is adequate compared to limit equilibrium method [19]. However, because the relation between the macroscopic strength parameters in the continuum method and mesoscopic strength parameters in the DEM is not always the linear relation, the application of the strength reduction method which is often used in the continuum method is difficult to be adopted to DEM with a reasonable demonstration. 

The reduced mesoscopic parameter in DEM, which corresponds to the cohesion c in FEM is the contact bond strength between the particles. According to the result of Zhou, the relation between the contact bond strength between the particles and the cohesion is linear relation. This relation can be described as [20]:

(23) $$K=\frac{NBS}{SBS}$$

(24) $$\frac{c}{SBS}=\alpha_{1}+\beta_{1}\ln\left(K\right)+\gamma_{1}\ln\left(\frac{k_{n}}{k_{s}}\right)$$

where $\alpha_{1}$, $\beta_{1}$, and $\gamma_{1}$ are constants if all the mesoscopic parameters in DEM have been set. It indicates that if NBS and SBS is reduced by the same proportion, the relation between the macroscopic parameter cohesion c and mesoscopic parameters contact bond strength NBS and SBS are linear, which make the strength reduction method in DEM possible.

However, the relation between the mesoscopic parameter “friction coefficient of ball surface μ“ and the macro parameter “the tangent value of internal friction angle tanφ” is not always linear relation. The macro friction f has two parts: the mesoscopic friction at the particle surface and the engagement force due to the roughness of the macro surface. It is not appropriate to adopt the mesoscopic friction as the friction for calculating the anti-sliding force. According to the result of Zhou, the relation between the mesoscopic strength parameter $\mu_{p}$ and the macroscopic parameter φ is as follows:

(25) $$\varphi =\alpha_{2}+\beta_{2}\frac{k_{n}}{k_{s}}+\gamma_{2}\ln\left(\mu_{p}\right)$$

where $\alpha_{2}$, $\beta_{2}$, and $\gamma_{2}$ are constants if all the mesoscopic parameters in DEM has been set. It indicates that this relation is nonlinear relation. Therefore, reducing the mesoscopic strength parameter NBS, SBS and μ linearly is not reasonable and cannot obtain a result that is comparable with the result of strength reduction method of FEM.

The reduced mesoscopic parameter in the calculating method raised in this paper which corresponds to the cohesion c in FEM is the contact bond strength NBS and SBS. Its reasonability is demonstrated in the section above. The reduced parameter in this article corresponding to the tangent values of internal friction angle tanφ in FEM is also tanφ. The corresponding equation is:

(26) $$f=\tan\varphi F_{n}^{\prime }S^{\prime }$$

$F_{n}^{\prime }$ is the normal force in the preset sliding circle surface in DEM. $S^{\prime }$ is the tangential direction of the preset sliding circle surface. tanφ is obtained from the calibration of the macroscopic parameter from virtual biaxial test in DEM. Obviously, the computing method is the same as computing method adopted in FEM. the reduced parameter of both of the methods is tanφ, which is consistent with limit equilibrium method.

### 5.3. The Comparsion with Gravity Increase Method of DEM

Another method to calculate the safety factor is gravity increase method which has been applied to solve the slope engineering problems in DEM [3,4]. Its detailed idea is increasing the acceleration of gravity successively until the failure of the slope. By defining $g_{0}$ as the gravitational acceleration in the initial state and $g_{0}^{\mathrm{trial}}$ as the gravitational acceleration at failure, the safety factor ω is defined as the following, according to Li et al. [21]:

(27) $$\omega =\frac{g_{0}^{\mathrm{trial}}}{g_{0}}$$

It is a method which calculates the safety factor by increasing the normal force and shear force at the same time until the failure of the slope, according to its definition:

(28) $$1=\frac{\int_{0}^{l}\left(c+\omega \sigma \tan\varphi \right)dl}{\omega \int_{0}^{l}\tau dl}=\frac{\int_{0}^{l}\left(\frac{c}{\omega }+\sigma \tan\varphi \right)dl}{\int_{0}^{l}\tau dl}$$

This overload coefficient is the safety factor on the condition of only reducing the cohesion c actually [22]. Moreover, the enlarged gravity may change the shape of the slope and the slope angle may decrease, which causes the inaccuracy of and the slope. It is more reasonable to reduce the cohesion and internal friction angle by the same proportion.

### 5.4. The Comparison with Slip Circle Method

In order to get spatial distribution of the slope stability except the critical sliding surface, the method preset the sliding surface such as the slip circle method. By reducing the mesoscopic strength parameter of the edge weight in the preset sliding surface, the safety factor of all the sliding surfaces can be calculated. This method considered the impact of the softening and the deformation in the granular assemblies compared with the slip circle method. Its result is consistent with the result of limit equilibrium method.

### 5.5. The Softening and the Instability Criterion

The strain softening can be classified into three kinds and the major kinds are damage softening and shearing dilatation softening [22]. For cohesive soil, the softening type is mainly damage softening which is the failure of the soil structure and the decrease of the cohesion. For sandy soil, the soften type is mainly shearing dilatation softening which is the decrease of internal friction angle caused by the increase of porosity.

In DEM, the damage softening of cohesive soil is realized by the break of bond when the contact force reaches the contact strength, which has been realized by the computing method raised in this article. The impact of dilatation softening is not as large as the damage softening in cohesive soil. The dilatation softening occurs when there is a certain amount of shear displacement. The result will be more adequate if this method considers the dilatation softening. In Section 4, if the dilatation is considered, the result is closer to the traditional method [23,24,25,26].

In the strength reduction method, the strength parameter is reduced to make the slope reach instability. The corresponding reduction coefficient is defined as safety factor. In general, the continuum method such as FEM adopts that the softening area cut through the section of the slope from slope toe to slope crest as the instability criterion.

This paper also adopts the softening area cut through the section of the slope as the instability criterion. For cohesive soil, the strength is dominated by the cohesion. Therefore, this paper adopts the bond break as the index of softening. When the number of the bonds in the edge sequence that is judged to break exceeds to a certain extent, the preset surface is judged to reach instability state and the corresponding reduction factor is the safety factor of the corresponding sliding surface. It is consistent with the strength reduction method of continuum method.

## 6. Conclusions

Among the numerical methods for simulating granular material, DEM is a kind of discontinuous mechanics method. Compared with other continuous mechanics methods, DEM has the advantage that it simulates the mesoscopic property of granular materials by the random configuration of the particles, which is closer to the property of the granular material [27,28]. Therefore, the slope analysis in DEM is necessary for researching on the property of the granular slope further. Its distinctive mesoscopic constitutive model makes some previous analysis methods based on the macro property of the material unfit for DEM. This paper raised a slope analysis method from mesoscopic scale to explore the mesoscopic topological structures and properties of the slope.

The analysis method of cohesive soil slope stability in this paper is based on the characterization method of macro contact. This characterization method has clear physical basis and is quantifiable in math. In physics, for two-dimensional granular assemblies, the shape of particle contact is consistent with the link of two adjacent voids. The macro-contact is a particle-contact sequence which satisfies the constraint condition of 1-manifold. The mathematical quantification is on the basis of graph theory. The particle contact and macro contact is characterized by the edge and edge sequence in which the particle contacts connect each other in the form of end-to-end respectively. With clear physical basis and simple mathematical operability, this characterization can integrate the originally discrete particle-contacts to continuous macro contacts, bridging the gap between the mesoscopic and macro scales of granular materials.

In DEM, neither the reduction of mesoscopic parameters nor the increase of the gravity acceleration can obtain a result that is comparable to the traditional methods. The analysis method of cohesive soil slope stability in this paper corresponds to the strength reduction method of FEM. This new analysis method needs to reduce the edge weights in the abstracted graph rather than to reduce mesoscopic strength parameters in DEM. The relation between the reduced mesoscopic parameter in the abstracted graph and the reduced macroscopic strength parameter “tanφ” and “c” in FEM is linear relation, which can get the result comparable with other traditional methods.

This paper raised a method that unites the mesoscopic characteristic and macro property of the granular material. It analyzes the cohesive granular slope stability from a mesoscopic perspective and can get the macro spatial distribution of the safety factor of the cohesive granular slope. It is a kind of slope analysis method from mesoscopic perspective so it can present more details of the mesoscopic property of the granular slope. For example, there are some discrete phenomena in the sectional distributions of the safety factor, which is consistent with the random configuration of the particles. In the respect of macro scale, the result is consistent with the traditional method, presenting good accuracy for predicting the cohesive slope stability. 

## Figures and Tables

**Table 1:** The parameters adopted to calculate the safety factor.

$F_{s^{\prime }}$	The shear force in the preset sliding surface
$F_{n^{\prime }}$	The normal force in the preset sliding surface
DIS	The distance from the circle center of the sliding surface to the contact point
S	The unit vector in the tangential direction of the particle contact surface
N	The unit vector in the normal direction of the particle contact surface
$S^{\prime }$	The unit vector in the tangential direction of preset sliding surface
$N^{\prime }$	The unit vector in the normal direction of preset sliding surface
f	The macro friction

**Table 2:** The macro parameters of the example.

Marco Parameters	Values
Soil unit weight γ	18.5 kN/m^3^
Cohesion c	20 kPa
Internal friction angle φ	15°
Elastic modulus $E^{\prime \prime }$	20 MPa
Poisson’s ratio ν	0.2

**Table 3:** The mesoscopic parameters of the example.

Mesoscopic Parameters	Values
Particle density $\rho_{p}$	2170 kg/m^3^
Particle radius R	0.05 m~0.1 m
Normal bond strength NBS	6 kPa
Shear bond strength SBS	6 kPa
Normal contact stiffness $K_{n}$	2.0 × 10^7^ N/m
Shear contact stiffness $K_{s}$	0.4 × 10^7^ N/m
Friction coefficient of the particle surface $\mu_{p}$	0.1
